# Infection Parameters in the Sand Fly Vector That Predict Transmission of *Leishmania major*

**DOI:** 10.1371/journal.pntd.0001288

**Published:** 2011-08-23

**Authors:** Lisa W. Stamper, Rachel L. Patrick, Michael P. Fay, Phillip G. Lawyer, Dia-Eldin A. Elnaiem, Nagila Secundino, Alain Debrabant, David L. Sacks, Nathan C. Peters

**Affiliations:** 1 Laboratory of Parasitic Diseases, National Institute of Allergy and Infectious Diseases, National Institutes of Health, Bethesda, Maryland, United States of America; 2 Biostatistics Research Branch, National Institute of Allergy and Infectious Diseases, National Institutes of Health, Bethesda, Maryland, United States of America; 3 University of Maryland Eastern Shore, Princess Anne, Maryland, United States of America; 4 Division of Emerging and Transfusion Transmitted Diseases, Office of Blood Research and Review (OBRR), Center for Biologics Evaluation and Research (CBER), Food and Drug Administration, Bethesda, Maryland, United States of America; Lancaster University, United Kingdom

## Abstract

To identify parameters of *Leishmania* infection within a population of infected sand flies that reliably predict subsequent transmission to the mammalian host, we sampled groups of infected flies and compared infection intensity and degree of metacyclogenesis with the frequency of transmission. The percentage of parasites within the midgut that were metacyclic promastigotes had the highest correlation with the frequency of transmission. Meta-analysis of multiple transmission experiments allowed us to establish a percent-metacyclic “cutoff” value that predicted transmission competence. Sand fly infections initiated with variable doses of parasites resulted in correspondingly altered percentages of metacyclic promastigotes, resulting in altered transmission frequency and disease severity. Lastly, alteration of sand fly oviposition status and environmental conditions at the time of transmission also influenced transmission frequency. These observations have implications for transmission of *Leishmania* by the sand fly vector in both the laboratory and in nature, including how the number of organisms acquired by the sand fly from an infection reservoir may influence the clinical outcome of infection following transmission by bite.

## Author Summary

Many infectious diseases are initiated when pathogenic organisms are deposited into the skin of the human host by the bite of an insect. In the case of the parasite *Leishmania*, the causative agent of Leishmaniasis, factors associated with the bite of the infected sand fly vector influence infection outcome, suggesting that this is the most relevant means to initiate disease in experimental hosts. However, transmission frequency, and the dose of parasites delivered when transmission occurs, can vary enormously both within and between populations of experimentally infected flies. This variability represents a major obstacle to the widespread use of sand flies to study Leishmaniasis. We identified a parameter of *Leishmania* infection in the sand fly vector that predicts the degree of transmission competence within a group of experimentally infected flies. We also identified environmental and biological factors that influence transmission frequency. This information will make experiments relying on infected sand fly challenge more manageable, thereby increasing the likelihood that infected sand fly challenge, rather than needle challenge, will be used in future experimentation. Lastly, we demonstrated that the number of organisms acquired by the sand fly can influence subsequent sand fly infection intensity, and that this infection intensity has implications for disease outcome.

## Introduction

Experimental transmission of the etiological agents of vector-borne, parasitic diseases such as malaria, filariasis, trypanosomiasis and the leishmaniases, by the natural vector is the most relevant biological means to study the initiation and outcome of infection in experimental hosts. In the case of the protozoan parasite *Leishmania*, sand flies become infected when they obtain a blood meal from an infected mammallian host. Once inside the sand fly gut, parasites transform from the intracellular amastigote stage to the extracellular promastigote stage. Parasites then undergo a maturation process over the course of 1–2 weeks that involves escape through the peritrophic membrane surrounding the bloodmeal, attachment to the midgut wall, and migration to the anterior midgut and foregut. Anterior migration is accompanied by differentiation of the parasite into the non-dividing metacyclic promastigote, which is the infectious form that is deposited in the skin during a second or subsequent feeding attempt [1]–[3].

Experimental infection of vertebrate hosts with *Leishmania* have only rarely been initiated using natural sand fly transmission. These few experiences have nonetheless revealed significant differences in disease outcome and host response to sand fly versus needle inoculation of parasites [4]–[6]. Most critically, mice vaccinated with a killed *Leishmania* vaccine are protected against needle challenge but not against parasites that are transmitted by sand fly bite. [5], [6]. These findings reinforce a series of studies demonstrating that needle injection of parasites with components of sand fly saliva or with promastigote secretory gel, both of which may be egested by infected sand flies, enhances disease [4], [7]–[11].

Experimental transmission of *Leishmania* by infected sand flies presents several challenges that seriously undermine the practicality and physiologic relevance of experiments intended to test infection outcomes following “natural” exposure to the bite or bites of a single infected sand fly. The development of transmissible infections can vary enormously both within and between populations of infected flies [4], [12]–[15]. Thus a large number of animal replicates and/or infected sand flies per animal are typically used to insure that a sufficient number of animals receive an infectious challenge, and to account for the wide variation in parasite dose delivered by individual flies [6], [16]. The goal of the studies reported here is to identify parameters of *Leishmania* infections within the sand fly vector that correlate with successful experimental transmission of parasites to the mouse dermis. This information will not only improve our understanding of host-vector-pathogen interactions, but will permit predictions as to the degree of transmission competence within a group of experimentally infected flies so that experiments relying on sand fly challenge will become more manageable and better reflect the conditions of natural exposure.

## Methods

### Mice

Female BALB/c and C57BL/6 mice were purchased from Taconic Farms. Mice were 6–10 weeks in age at the time of exposure to sand flies. All mice were maintained in the National Institute of Allergy and Infectious Diseases animal care facility under specific pathogen-free conditions.

### Parasites

*Leishmania major* RYN Strain (*L. m.* RYN) was isolated from a lesion biopsy of a laboratory worker accidentally exposed to *Lutzomia longipalpis* sand flies that were experimentally infected with a strain of *L. major* (WR2885) originating in Iraq and isolated at the Walter Reed Army Institute of Research. A clone was obtained by limiting dilution and used to infect *P. duboscqi* sand flies. The *L. major* FV1 (Friedlin) strain is from the Jordan Valley, NIH/FV1 (MHOM/IL/80/FN). A stable transfected line of *L.m.* RYN promastigotes expressing a red fluorescent protein was generated as described previously [12]. The resulting parasite is referred to as *L.m.* RYN-RFP. All parasites were grown *in-vitro* at 26°C in medium 199 supplemented with 20% heat-inactivated FCS (Gemini Bio-Products), 100 U/ml penicillin, 100 µg/ml streptomycin, 2 mM L-glutamine, 40 mM Hepes, 0.1 mM adenine (in 50 mM Hepes), 5 mg/ml hemin (in 50% triethanolamine), and 1 mg/ml 6-biotin. The *L.m.* RYN-RFP line was grown in the presence of 50 µg/ml Geneticin (G418) (Sigma).

### Sand Fly Infection and Determination of Sand Fly Infection Parameters

Two-to-four day old *Phlebotomus duboscqi* females were obtained from a colony initiated from field specimens collected in Mali. Flies were infected by artificial feeding through a chick skin membrane on heparinized mouse blood (drawn intracardially from BALB/c mice), penicillin (100 U/ml), streptomycin (100 ug/ml) and *L. major* promastigotes. Sand fly infection ‘dose’ refers to the concentration of *L.major*. promastigotes per ml of blood upon which flies were allowed to feed. A fully engorged female *P. duboscqi* sand fly takes a blood meal of approximately 0.2–0.3 ul. Blood engorged flies were separated and maintained at 26°C and 75% humidity and were provided 30% sucrose *ad libitum*. After 13–14 days, 9–10 flies per experimental group were anesthetized with CO_2_, killed in 5% soap solution, and whole midguts, including the crop, were dissected and transferred into tubes containing 25 µl 1× PBS. The guts were macerated briefly using a plastic pestle, then spun twice at 800 rpm for 1 minute to remove the debris. A 10-µl sample of the supernatant was counted under a hemocytometer and the numbers of metacyclic promastigotes, non-metacyclic forms, and total parasite number, as determined by morphology and movement, were counted.

### Exposure of Mice to Infected Sand Flies

*Leishmania* infections were allowed to mature for 14–16 days within the sand fly midgut. One day before transmission the sucrose diet was removed. On the day of transmission, 4–5 flies were transferred to small plastic vials (volume 12.2 cm^2^, height 4.8 cm, diameter 1.8 cm) covered at one end with a 0.25-mm nylon mesh. Mice were anesthetized by intraperitoneal injection of 30 ul of ketamine/xylazine (100 mg/ml). Specially designed clamps were used to bring the mesh end of each vial flat against the ear, allowing flies to feed on exposed skin for a period of 2–3 hours in the dark at 23°C and 50% humidity. In some experiments, sand flies were first induced to oviposit by placing blood-fed flies in a plaster-lined pot on day 5 after infection. On day 8–9 following infection flies were returned to paper cups and a diet of 30% sucrose. In some experiments, transmission occurred at 23°C and 30% humidity or 26°C and 75% humidity. Following exposure to the ear, the number of flies per vial with a blood meal was determined using a dissecting microscope. The average number of flies per vial with or without a blood meal was used to determine the potential influence of feeding intensity on transmission frequency and parasite load. Feeding intensity among different groups of infected flies was the same unless noted otherwise.

### Determination of Lesion Size and Parasite Load

Following exposure to infected sand flies, ear lesion diameters were measured (in mm) weekly for 5–6 weeks. Ears with more then one lesion are reported as total lesion diameter per ear. 5–7 weeks following transmission, mice were euthanized, ears removed and each ear was washed in 70% ethanol, separated into two sheets, and incubated at 37°C for 90 minutes in 1 ml of DMEM with 40 mM Hepes and 0.2 mg/ml Liberase. The ear sheets were then ground in a Medimachine (Becton Dickenson). The tissue homogenate was added to 10 ml RPMI media containing 0.05% DNAse, filtered using a 70 um-pore-size cell strainer, spun-down for 10 mins at 1500 rpm, re-suspended in parasite growth medium and serially diluted in a 96-well flat-bottom microtiter plate, overlaying 100 ul onto 50 ul of NNN medium containing 20% defibrinated rabbit blood. The number of viable parasites in each ear was determined from the highest dilution at which promastigotes could be grown after 7 to 10 days of incubation at 26°C.

### Statistics

**Figure 1:**
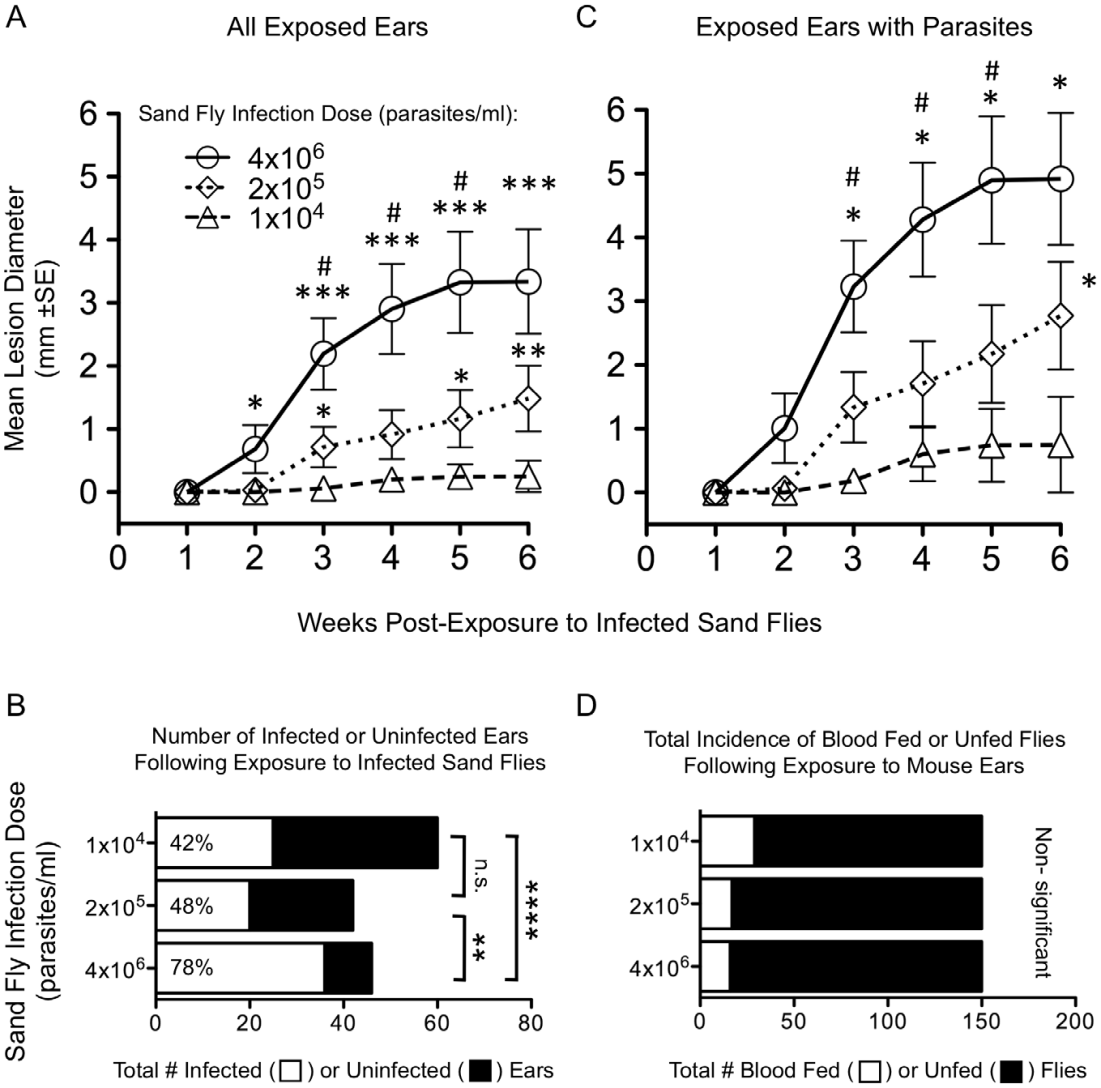
Sand fly infection dose alters frequency of transmission and disease severity. *(A)* Mean lesion diameters (±1 SE, n = 28–36 ears per group) after exposure to *P. duboscqi* sand flies infected with the indicated dose of *L.m.*. Asterisks indicate significant differences versus the 1×10^4^ group (*, 0.014<p<0.043; **p = 0.002; ***p<0.001). Hash symbols indicate significant differences versus the 2×10^5^ group (#, 0.016<p<0.043). Data is from 2, pooled experiments. *(B)* Representation of the total incidence of infected (open bar) versus uninfected (filled bar) ears in mice exposed to sand flies infected with the indicated dose of *L.m* (n = 42–60 ears/group) at 7 weeks post-transmission. **, p = 0.005 [common odds ratio 4.07, 95% CI (1.55–10.70)]; ****, p<0.0001 [common odds ratio 15.83. 95% CI (3.61–69.50)]; n.s. = non-significant, p = 0.457 [common odds ratio 1.50, 95% CI (0644–3.48)]. The percentage within the open, infected, bar represents the frequency of transmission. Data is from 4, pooled experiments. (*C*) Mean lesion diameters (±1 SE, n = 12–19 ears per group) from those mice in panel *(A)* that were positive for *L.m.* Asterisk indicate significant differences versus the 1×10^4^ group (*, 0.015<p<0.042). Hash symbols indicate significant differences versus the 2×10^5^ group (#, 0.028<p<0.052). *(D)* Representation of the total incidence of blood meals in flies infected with the indicated concentration of *L.m.* per ml of blood and exposed to mouse ears. Data is from 2, pooled, experiments employing 5 flies per vial and a total of 30 ears/group.

**Figure 2:**
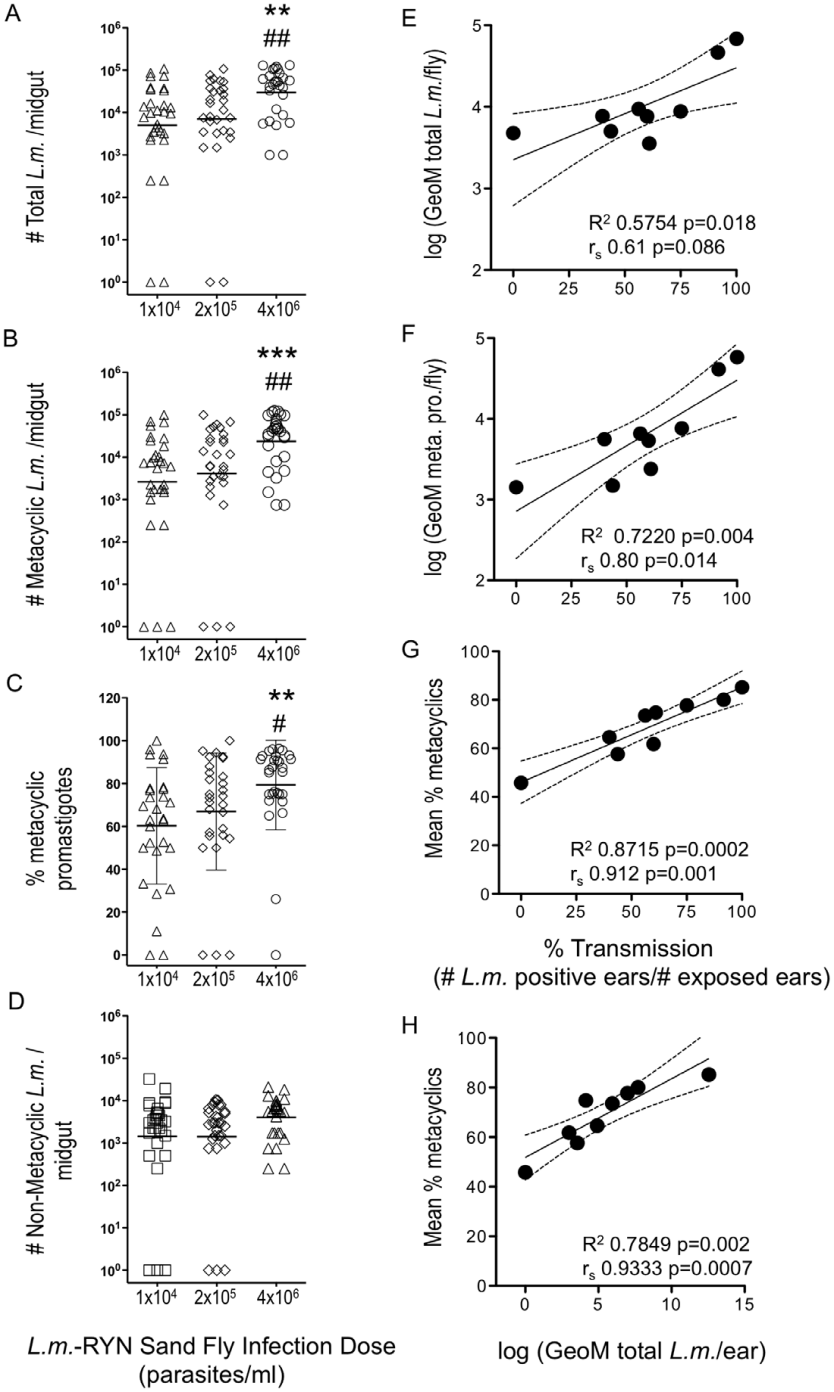
Relationship among pre-exposure sand fly infection parameters and sand fly infection dose, percent transmission, or parasites per exposed ear. *(A)* total number of parasites, *(B)* total number of metacyclic promastigotes, *(C)* percent metacyclic promastigotes, or *(D)* total number of non-metacyclic promastigotes per midgut in individual flies infected with the indicated dose of parasites (n = 9–10 flies/dose/experiment). Asterisks indicate significant differences versus the 1×10^4^ group (**, 0.0012<p<0.0026 and ***, p = 0.0007). Hash symbols indicate significant differences versus the 2×10^5^ group (#, p = 0.023, and ##, p = 0.007). *(E–G)* Pooled data showing percent *L.m.* transmission to ears (n = 10–20 ears per data point) exposed to 5 flies infected with one of three doses of *L.m.* indicated in (A) as a function of the Geometric Mean *(E)* total number parasites, *(F)* total number metacyclic promastigotes, or *(G)* mean percent metacyclic promastigotes in the corresponding pre-exposure sample of flies. *(H)* Geometric mean total parasites per exposed ear as a function of percent metacyclics. Solid line represents the linear regression line ± the 95% C.I. (dashed lines). Data is from 3, pooled, experiments.

**Figure 3:**
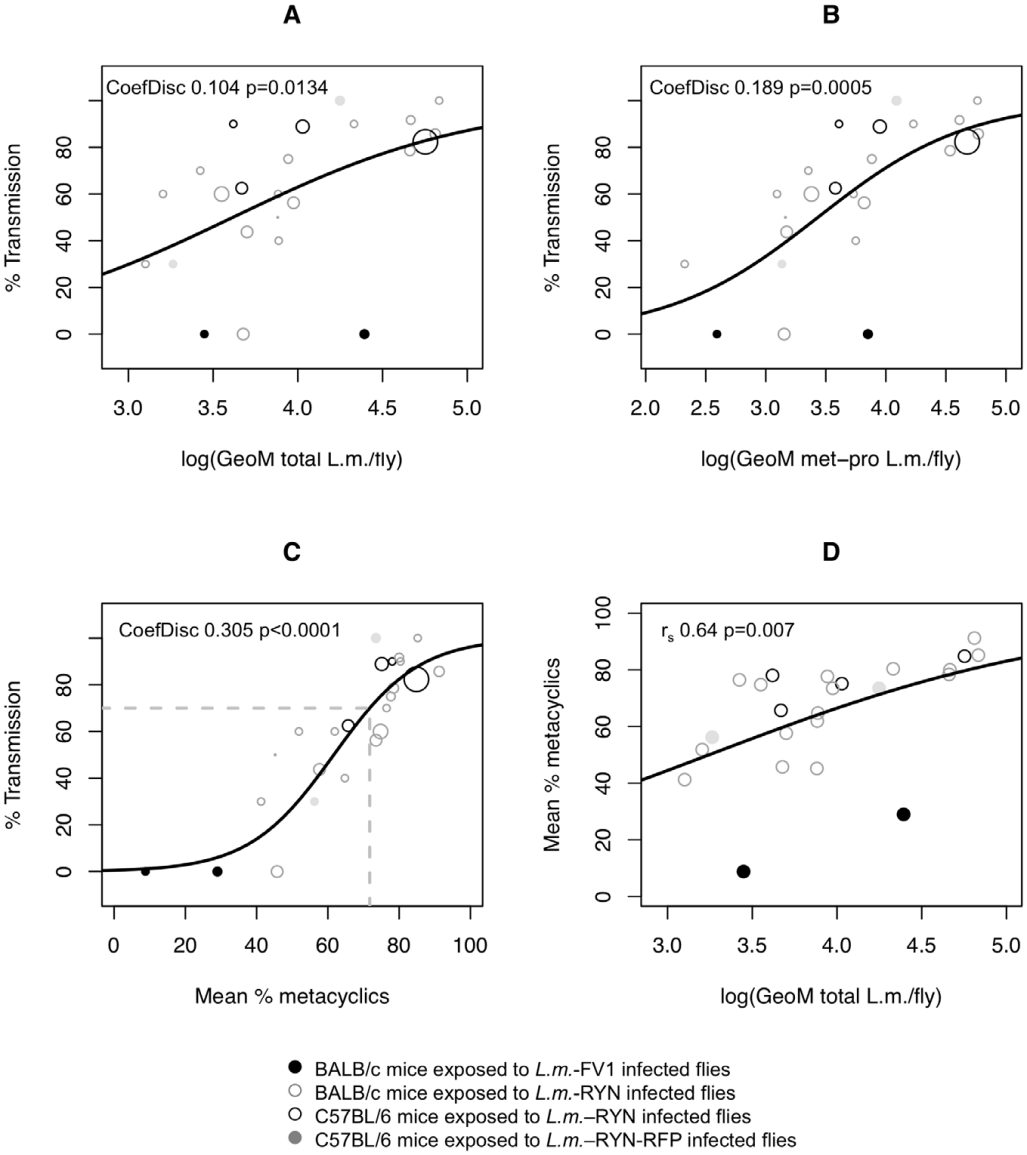
Meta-Analysis of the relationship between pre-exposure sand fly infection parameters and percent transmission. Pooled data showing percent *L.m.* transmission to ears (n = 2–34 ears per data point, diameters of points proportional to n) exposed to 5 flies as a function of the Geometric Mean *(A)* total number parasites, *(B)* total number metacyclic promastigotes, or *(C)* mean percent metacyclic promastigotes in the corresponding pre-exposure sample of flies. *(D)* Geometric mean total parasites per mid-gut as a function of percent metacyclics. Solid black line represents either a standard (A–C) or a nonlinear least squares (D) logistic regression model. Data is from 16, pooled, experiments employing 24 different groups of infected flies exposed to a total of 314 ears. CoefDisc is coefficient of discrimination, r_s_ is Spearman r, p is test of significant effect (A–C from logistic regression, D from test on Spearman's correlation).

To compare two groups with continuous responses (e.g., lesion diameters, parasite loads), we used the Mann-Whitney test, stratified by experiment in order to allow pooling of data where appropriate. To compare two groups with binary responses (e.g., presence of infection, presence of blood meal) we used either Fisher's exact test for single experiments or the Mantel-Haenszel chi-squared test with continuity correction for combined experiments, with effects measured by odds ratios, for example see Figure 1B. We used Spearman's correlation (reported as r_s_) to compare pairs of continuous responses. The Meng, Rosenthal, Rubin (MRR) method [17] with Holm's adjustment for multiple comparisons [18] was employed to test for significant differences between Spearman correlations. Linear regression was used in Figures 2 E–H. To model percent transmission in Figure 3, we used logistic regression with a quasi-likelihood model that allows over dispersion in the variance estimate that may be caused by lack-of-fit of the model. In Figure 3D we used a nonlinear least squares fit of a logistic model. All p-values are two-sided. Statistical calculations were done in Graphpad PRISM 5.0c (www.graphpad.com) or R 2.12.0 (www.r-project.org) with the coin package [19].

### Ethics Statement

All animal experiments were performed under an Animal Study Protocol approved by the NIAID Animal Care and Use Committee under guidelines established by the Animal Welfare Act and the PHS Policy on Humane Care and Use of Laboratory Animals.

## Results

### Identification of *Leishmania major* Infection Parameters in Experimentally Infected *Phlebotomus duboscqi Neveu-Lemaire* Sand Flies That Correlate with Transmission

For the purpose of employing experimental sand fly challenge to study the host response to *Leishmania* infection under conditions that best reflect those of natural transmission, the ideal exposure would be to a single infected fly. We previously reported, however, that of the 301 *L.m.* FV1 infected *P. duboscqi* sand flies exposed singly to the mouse ear dermis, only 58 or 19% transmitted parasites. This frequency increased to only 25% (18/72) when the analysis was confined to infected flies that successfully acquired a second blood meal from an exposed mouse [12]. In order to achieve an acceptable rate of transmission to exposed animals, while at the same time trying to avoid an unnaturally high exposure to the immunomodulatory effects of sand fly bites, in our recent studies we have typically exposed the ear dermis to 4–5 infected sand flies [6], [16]. Employing 4–5 infected flies as a constant, we have nonetheless noted a wide range in the frequency of successful transmissions, from 0% to 100%, varying as a function of the strain of parasite used, its culture history, and the concentration of organisms used to establish the infective blood meal.

Figure 1A–C shows the outcome of *L. major* infections in BALB/c mice transmitted by flies with midgut infections initiated experimentally with varying doses of *L.m.* RYN, a recent primary clinical isolate of *L. major*. We approximate the initial dose of parasites acquired by individual sand flies in our experimental system to be 2–3, 40–60, or 800–1200 parasites/fly depending upon whether the flies are fed on blood containing 1×10^4^, 2×10^5^ or 4×10^6^ parasites/ml, respectively. BALB/c mouse ears exposed to flies infected with increasing doses of parasites developed larger lesions when compared with ears exposed to flies infected with lower doses (Fig. 1A). Employing the infection status of each exposed ear to determine transmission success revealed that flies infected with either 2×10^5^ or 1×10^4^ per ml of blood were significantly less likely to transmit than those flies infected with the 4×10^6^ dose (Fig. 1B). Exposed ears that do not have lesions include both uninfected ears and infected ears that have yet to present with lesions. Analysis of only those ears that were successfully infected revealed that ears exposed to flies infected with 4×10^6^ parasites per ml of blood had significantly larger lesions starting at 3 weeks post-transmission (Fig. 1C). The difference in lesion sizes in Figure 1A versus 1C illustrates the need to confirm the presence or absence of parasites when interpreting lesion data following infected sand fly challenge. The observed differences in lesion size could not be accounted for by the inability of flies infected with lower doses of parasites to feed. The total number of flies with or without a blood meal following exposure to mouse ears revealed no significant differences between the groups of flies infected with different doses of parasites (Fig. 1D).

In order to identify parameters of *Leishmania* infections within the sand fly that correlate with experimental transmission, on day 13–14 of the infection, 9–10 flies from each group used in the transmission experiments described above were dissected and the viable promastigotes representing the various developmental stages were discriminated and counted on a hemocytometer. Infections initiated with 2×10^5^ or 1×10^4^ parasites/ml of blood generated *Leishmania* infections with lower numbers of total parasites (Fig. 2A), lower numbers of metacyclic promastigotes per fly (Fig. 2B), and significantly decreased percentages of metacyclic promastigotes (Fig. 2C).

Sand flies become infected in nature with tissue- or blood-derived amastigotes. In contrast, we employed culture-derived promastigotes mixed with mouse blood to initiate our sand fly infections. This was done to minimize the manipulation of the parasite following isolation from the original clinical biopsy. Sand fly infections initiated with *L.m.* RYN promastigotes, or amastigotes generated following passage through mice, revealed no significant difference in the percentage of metacyclic promastigotes, 91.2±6 versus 85.0±15, respectively, p = 0.67, n = 9 flies per group, suggesting the high frequency of metacyclics observed was not an artifact of employing promastigotes for sand fly infection.

Comparison of the GeoM of the total number of parasites per fly with percent transmission (n = 10–20 ears per dose per experiment, total n = 148 ears) revealed a weak linear trend (R^2^ 0.5754 p = 0.018) but a non-significant correlation (Spearman r 0.617; p = 0.086) (Fig. 2E). In contrast, an increase in the number of metacyclic promastigotes or the percentage of metacyclic promastigotes returned higher rates of correlation with percent transmission (Spearman r 0.80; p = 0.014 and 0.91; p = 0.001, respectively) (Fig. 2, F and G). The percentage of metacyclic promastigotes returned the highest Spearman r value for correlation (0.91 versus 0.80 (total met-pro/fly) and 0.61 (total parasites/fly)), and this was also reflected in the correlation between percent metacyclics and the geometric mean of the parasite loads detected in ears exposed to the different groups of flies, (Spearman r 0.933; p = 0.0007) (Fig. 2H). These results suggest that by determining the total number and percentage of metacyclic promastigotes from a sample of a larger group of experimentally infected flies, the frequency of transmission can be accurately predicted.

### A Meta-Analysis of Sand Fly Infection Parameters and Transmission Frequency

We analyzed data in which mice were exposed to the bites of 24 different groups of experimentally infected flies in 16 different experiments comprising 314 exposed ears, including those experiments in which flies were infected with different doses of *L.m.* RYN parasites shown in Figures 1 and 2, as well as flies infected with a poorly transmitted, culture-adapted, line of *L.m.* FV1. The correlation between percent transmission and mean percent metacyclics (Spearman r 0.85; 95% C.I. 0.681–0.937) (Fig. 3C) was significantly stronger than percent transmission and total parasite load (Spearman r 0.56; 95% C.I. 0.191–0.791) (Fig. 3A), p_H_ = 0.0065, or percent transmission and total number of metacyclic promastigotes (Spearman r 0.74; 95% C.I. 0.478–0.886) (Fig. 3B), p_H_ = 0.023. In addition, percent metacyclics returned the lowest p-value (p<0.0001) as compared to total parasite load (p = 0.013) or total number of metacyclic promastigotes (p = 0.0005) when the different parameters were tested for their ability to predict transmission using a logistic regression model. Thus, the proportion of midgut promastigotes that have differentiated to metacyclic forms is the best predictor of transmission success based on the meta-analysis of multiple data sets in which ears were exposed to 5 infected flies.

Applying a logical regression model to the data in Figure 3C, it can be predicted that in order to achieve ≥70% transmission as an arbitrary frequency sufficient to conduct experiments (dashed horizontal line in Figure 3C), the percent metacyclics would need to be 71.7% (95% confidence interval 67.3–75). Groups of flies with frequencies of metacyclic promastigotes above 71.7% transmitted parasites to an average of 82% (SD±14) of exposed ears, while those groups with below 71.7% metacyclic promastigotes transmitted parasites to an average of 34% (SD±26) of exposed ears. In addition, applying a cut-off of 71.7% metacyclic promastigotes would have excluded the use of 11 of the 13 groups of sand flies that transmitted to <70% of exposed ears. Therefore, a cutoff of 71.7% metacyclic-promastigotes can be employed in future experiments to predict if a group of experimentally infected flies, employing 5 flies per animal, should be used for transmission.

Our meta-analysis also revealed a correlation between total parasite numbers and mean percent metacyclics (Spearman r 0.64; p = 0.007; 95% C.I. 0.314–0.835) (Fig. 3D). These results add to our previous findings in which an increase in the number of parasites in a single fly was associated with an increase in the frequency of metacyclic promastigotes [12].

### Additional Factors Influencing the Transmission of *L. major* by Experimentally Infected *P. duboscqi* Sand Flies

**Figure 4:**
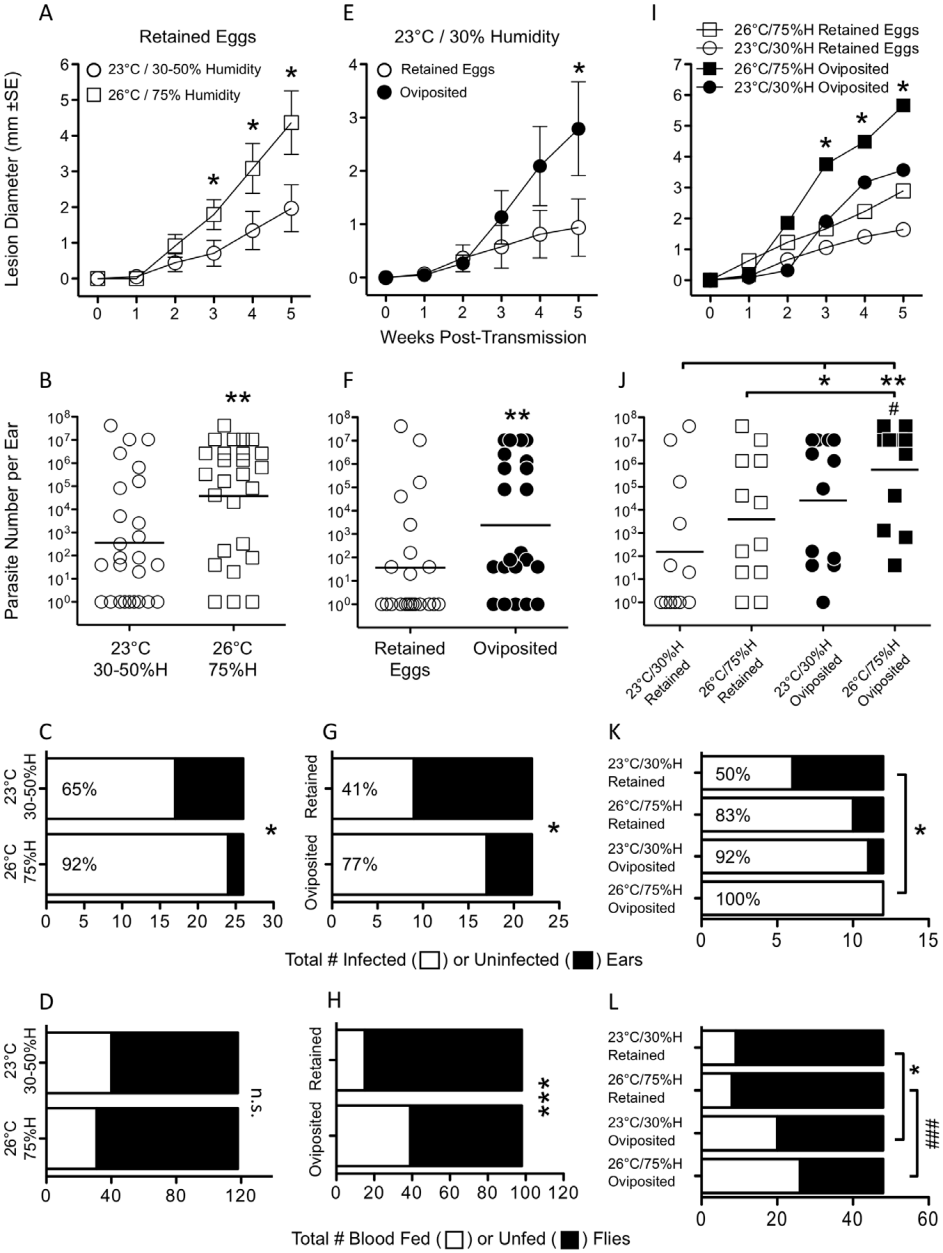
Environmental conditions and sand fly oviposition status influence transmission. Ear lesion diameters *(A, E, and I)*, parasite loads per ear *(B, F, and J)*, number of infected or uninfected ears *(C, G, and K)*, and presence or absence of a blood meal in the sand fly *(D, H, and L)* following exposure of BALB/c mice to *L.m.*-RYN infected sand flies (4×10^6^ infection dose) under the indicated environmental conditions (*A–D*, n = 26 ears/group; *E–H*, n = 22 ears/group; *I–L*, n = 12 ears/group). Asterisk indicate significant differences versus the 23°C/30% Humidity and Retained Eggs group (*, 0.014<p<0.056; **, 0.008<p<0.010; ***, p = 0.0002). Hash symbols indicate significant differences versus the 26°C/75% Humidity and Retained Eggs group (#, p = 0.033; ###, p = 0.0002) In *(C)* common odds ratio 0.125, 95% C.I. (0.020–0.764); in *(D)* n.s., p = 0.246 [common odds ratio 0.683, 95% C.I. (0.386–1.21)]; in *(G)* common odds ratio 0.175, 95% CI (0.043–0.722); in *(H)* common odds ratio 0.272, 95% CI (0.137–0.540), Data are from three experiments: (1) flies with retained eggs under different environmental conditions, (2) oviposited flies or flies with retained eggs under the same environmental condition and (3) a 2 by 2 factorial experiment that varies both environmental conditions and oviposition status. Plots *(A–H)* combine experiments 1 and 3, plots *(E–H)* combine experiments 2 and 3, and plots *(I–L)* are experiment 3 alone. In *(C, G, and K)* the number within the open, infected, bar represents the frequency of transmission.

We also explored the influence of environmental conditions and sand fly oviposition status on the transmission rate. Ears exposed to infected sand flies, with retained eggs, at 26°C and 75% humidity presented with significantly larger lesions, greater numbers of parasites, and were more likely to be infected (Fig. 4A–C) compared to ears exposed at 23°C and 30–50% humidity. Allowing infected flies to oviposit also resulted in increased lesion size, greater overall parasite loads, and a significantly increased rate of transmission following exposure to mouse ears at 23°C and 30% humidity (Fig. 4E–G). In order to assess if oviposition status and increased humidity and temperature have an additive effect on transmission we directly compared each of these conditions alone or in combination in one experiment (Fig. 4I–L). Exposure of ears to infected, oviposited, flies at 26°C and 75% humidity trended towards an additive effect over ears exposed to oviposited flies at 23°C and 30% humidity or flies that retained eggs at 26°C and 75% humidity. In addition, the combination of conditions resulted in significantly greater lesion sizes, larger parasite loads, and a higher frequency of transmission compared to infections initiated by infected flies that retained eggs at 23°C and 30% humidity. Flies that had oviposited were more likely to take a blood meal compared to flies with retained eggs (Odds ratio, 0.274, 95% C.I. 0.162–0.455, p<0.00001) (Fig. 4H). This result suggests flies feed more efficiently or pursue a second blood meal more aggressively after they have oviposited, and this may partially explain the enhanced rate of transmission by these flies (Fig. 4E–G). In contrast, enhanced transmission by infected flies with retained eggs under conditions of higher temperature and humidity (Fig. 4A–C) was not associated with an increase in their ability to acquire a blood meal (Odds ratio, 0.658; 95% C.I. 0.412–1.05, p = 0.078) (Fig. 4D). Therefore, oviposition status and environmental conditions at the time of exposure may influence the transmission of *Leishmania* by infected sand flies.

## Discussion

The use of sand flies to initiate experimental infection with *Leishmania* has revealed several important biological differences in the host response to infection and disease outcome as compared with needle inoculation [4]–[6]. However, the number of reports employing experimental transmission of *Leishmania* by bite, including evidence of viable transmitted organisms, is scant. This is due, at least in part, to the unpredictability of parasite transmission by sand fly bite, compounded by a shortage of information on the parameters of sand fly infection that influence transmission. In the studies reported here we identified a parameter of *Leishmania* infection within the sand fly, the percentage of metacyclic promastigotes, which best correlates with the subsequent frequency of parasite transmission to the mammalian host in an experimental setting. Meta-analysis of our extensive experience involving transmission attempts by 4–5 *L. major*-infected flies exposed to the mouse ear dermis permitted us to establish a threshold of metacyclic percentage in infected flies (71.7%) that reliably predicts an acceptable level of transmission success, arbitrarily defined as ≥70%.

The predictive value of the infection parameters reported here should help make experiments relying on sand fly challenge more manageable. In particular, it should prevent the use of populations of infected flies that are unlikely to transmit to a sufficient number of animals to interpret subsequent experimental results. This is of critical importance in experiments where the animals used for challenge, e.g. vaccinated mice, monkeys, or dogs, are valuable and limited.

While increasing the number of flies per animal could be used to compensate for flies harboring sub-optimal numbers of metacyclics, we would argue that over-exposure of the inoculation site to sand fly bites begins to substitute one form of non-physiologic exposure, i.e. needle challenge, for another. This raises the question as to whether the numbers of parasites per midgut and frequencies of metacylics achieved in our experimental system reflect what is found in naturally infected flies. Published data on the intensity and composition of *Leishmania* infections within wild-caught sand flies are rare. One study revealed parasite loads of between 10^1^ to 10^6^ parasites per fly that appeared to vary according to oviposition status [20], although this was no doubt also influenced by the stage of infection in the fly. Groups of laboratory-reared *P. duboscqi* flies with mature experimental *L. major* infections average between 10^2^ to 10^5^ parasites per midgut depending upon the dose of parasites used for infection [12], [15]. Similar results have been reported following experimental infection of *Lutzomyia longipalpus* with *L. infantum* (Lutz and Neiva) [13] and *Lu. Longipalpus* with *L. mexicana* [4]. As far as we are aware, there is no published data on the frequency of metacyclic promastigotes within infected wild caught flies. Therefore, we can only speculate that flies infected with *L.m.* RYN, which contain in the range of 10^3^–10^6^ total parasites per fly and high frequencies of metacyclic promastigotes, approximate the infection status of transmitting flies in the wild.

In nature, sand flies likely become infected with varying doses of parasites depending upon their feeding behavior and the concentration of parasites in the lesion or blood upon which they feed. We observed that groups of flies infected with larger initial doses of parasites had heavier infections, were more likely to transmit parasites during a second feeding attempt, and caused more severe disease when transmission occurred. This increased disease severity was likely due to a larger inoculum transmitted by the more heavily infected flies. In the single fly transmissions analyzed by Kimblin et al. [12], there was a direct correlation between the intensity of midgut infections and transmitted dose.

These results also suggest that the number of organisms picked up from an infection reservoir may have direct bearing on the severity of disease resulting from the bite of that infected fly. Thus, reservoir hosts, including humans for the anthroponotic forms of Leishmaniasis, with active infections containing large numbers of parasites may be more likely to give rise to heavily infected flies that will transmit more severe infections. The influence of transmitted dose on infection outcome has been difficult to study because it is impossible to control the number of parasites an individual sand fly deposits in the skin. Our results suggest that groups of flies infected with varying doses of parasites will deposit corresponding doses of parasites upon exposure to the dermis, leading to more or less severe disease.

Finally, we demonstrated a role for infected sand fly oviposition status and environmental conditions at the time of transmission in subsequent transmission frequency. These observations add to those of Rogers et al. [21] in which maturation of *L. mexicana* infections within *Lu. longipalpus* sand flies resulted in more persistent sand fly feeding behavior and enhanced transmission. Together, these experiences should facilitate studies of the host response to Leishmania infection under conditions of experimental sand fly challenge, already severely limited by the few laboratories that have sand flies available for study.

## References

[1] Sacks D, Kamhawi S (2001). Molecular aspects of parasite-vector and vector-host interactions in leishmaniasis.. Annu Rev Microbiol.

[2] Bates PA (2008). Leishmania sand fly interaction: progress and challenges.. Curr Opin Microbiol.

[3] Volf P, Hostomska J, Rohousova I (2008). Molecular crosstalks in Leishmania-sandfly-host relationships.. Parasite.

[4] Rogers ME, Ilg T, Nikolaev AV, Ferguson MA, Bates PA (2004). Transmission of cutaneous leishmaniasis by sand flies is enhanced by regurgitation of fPPG.. Nature.

[5] Rogers ME, Sizova OV, Ferguson MA, Nikolaev AV, Bates PA (2006). Synthetic glycovaccine protects against the bite of leishmania-infected sand flies.. J Infect Dis.

[6] Peters NC, Kimblin N, Secundino N, Kamhawi S, Lawyer P (2009). Vector transmission of leishmania abrogates vaccine-induced protective immunity.. PLoS Pathog.

[7] Titus RG, Ribeiro JM (1988). Salivary gland lysates from the sand fly Lutzomyia longipalpis enhance Leishmania infectivity.. Science.

[8] Belkaid Y, Kamhawi S, Modi G, Valenzuela J, Noben-Trauth N (1998). Development of a natural model of cutaneous leishmaniasis: powerful effects of vector saliva and saliva preexposure on the long-term outcome of Leishmania major infection in the mouse ear dermis.. J Exp Med.

[9] Rogers M, Kropf P, Choi BS, Dillon R, Podinovskaia M (2009). Proteophosophoglycans regurgitated by Leishmania-infected sand flies target the L-arginine metabolism of host macrophages to promote parasite survival.. PLoS Pathog.

[10] Rogers ME, Corware K, Muller I, Bates PA (2010). Leishmania infantum proteophosphoglycans regurgitated by the bite of its natural sand fly vector, Lutzomyia longipalpis, promote parasite establishment in mouse skin and skin-distant tissues.. Microbes Infect.

[11] Valenzuela JG, Belkaid Y, Garfield MK, Mendez S, Kamhawi S (2001). Toward a defined anti-Leishmania vaccine targeting vector antigens: characterization of a protective salivary protein.. J Exp Med.

[12] Kimblin N, Peters N, Debrabant A, Secundino N, Egen J (2008). Quantification of the infectious dose of Leishmania major transmitted to the skin by single sand flies.. Proc Natl Acad Sci U S A.

[13] Ranasinghe S, Rogers ME, Hamilton JG, Bates PA, Maingon RD (2008). A real-time PCR assay to estimate Leishmania chagasi load in its natural sand fly vector Lutzomyia longipalpis.. Trans R Soc Trop Med Hyg.

[14] Sadlova J, Price HP, Smith BA, Votypka J, Volf P (2010). The stage-regulated HASPB and SHERP proteins are essential for differentiation of the protozoan parasite Leishmania major in its sand fly vector, Phlebotomus papatasi.. Cell Microbiol.

[15] Myskova J, Votypka J, Volf P (2008). Leishmania in sand flies: comparison of quantitative polymerase chain reaction with other techniques to determine the intensity of infection.. J Med Entomol.

[16] Peters NC, Egen JG, Secundino N, Debrabant A, Kimblin N (2008). In vivo imaging reveals an essential role for neutrophils in leishmaniasis transmitted by sand flies.. Science.

[17] Meng X-L, Rosenthal R, Rubin DB (1992). Comparing correlated correlation coefficients.. Psycological bulletin.

[18] Wright SP (1992). Adjusted P-values for Simultaneous Inference.. Biometrics.

[19] Hothorn T, Hornick K, van de Wiel MA, Zeileis A (2008). Implementing a Class of Permutation Tests: The coin Package.. Journal of Statistical Software.

[20] Reale S, Torina A, Sole M, Calderone S, Piazza M (2008). Fluorescence-based detection of Leishmania infantum DNA in phlebotomus vectors.. Ann N Y Acad Sci.

[21] Rogers ME, Bates PA (2007). Leishmania manipulation of sand fly feeding behavior results in enhanced transmission.. PLoS Pathog.

